# ATDIOU: Arctangent Differential Loss Function for Bounding Box Regression

**DOI:** 10.3390/s26051545

**Published:** 2026-03-01

**Authors:** Qiang Tang, Hao Qiang, Yuan Tian, Xubin Feng, Wei Hao, Meilin Xie

**Affiliations:** 1 Xi’an Institute of Optics and Precision Mechanics of CAS, Xi’an 710119, China; 2 University of Chinese Academy of Sciences, Beijing 100049, China

**Keywords:** computer vision, object detection, bounding box regression, arctangent-differential function

## Abstract

Object detection is a fundamental task in computer vision. Bounding box regression (BBR) losses are critical to detector performance. However, evaluation measures that rely on the Intersection over Union (IoU) between the predicted and ground truth boxes are highly sensitive to positional deviations, which can hinder optimization. To alleviate this issue, we propose ATDIoU, a novel arctangent-differential loss for bounding-box regression. ATDIoU computes distance similarity between a predicted and a ground truth box by modeling the distances between their corresponding vertices as a two-dimensional arctangent differential distribution (ATD). This arctangent differential-based design mitigates bounding box drift and reduces sensitivity to localization errors. As a result, it guides the model to learn target positions more effectively. We evaluate ATDIoU by integrating it into YOLOv6 and conducting experiments on PASCAL VOC and VisDrone2019. The results demonstrate that ATDIoU yields improvements of 1.4% and 0.7% in mean average precision (mAP) relative to MPDIoU.

## 1. Introduction

Precise bounding box regression (BBR) is fundamental to object detection. As a core task in computer vision, object detection underpins a diverse array of applications, including autonomous driving, video surveillance, medical image analysis, and remote sensing. The accuracy of bounding box localization critically influences the performance of downstream tasks, such as instance segmentation, object tracking, and activity recognition. Modern detectors, including both one-stage and two-stage frameworks, typically use Intersection over Union (IoU)–based metrics or their variants to supervise the localization process [1,2,3,4,5,6,7,8]. Due to their intrinsic scale invariance and alignment with evaluation metrics, IoU and its derivative, IoU loss, remain the predominant choice for BBR.

Despite their efficacy in high-overlap scenarios, IoU-based losses present two significant limitations. First, they exhibit high sensitivity to spatial misalignment. In instances where the predicted and ground truth boxes are non-overlapping, the IoU metric yields a zero value, resulting in vanishing gradient. This phenomenon severely impedes model optimization, particularly during the initial training phases or when processing hard examples. Second, these losses demonstrate instability regarding boundary deviations. Slight positional shifts near edges can cause large fluctuations in overlap. Such instability destabilizes gradient updates and impedes convergence [9,10,11,12,13,14,15,16]. These deficiencies are particularly pronounced in tasks involving small objects or dense scenes. In these contexts, precise corner alignment is imperative, and the tolerance for error is negligible.

To mitigate these deficiencies, extensive research has focused on extending the standard IoU metric. A prevalent strategy involves augmenting the loss function with geometric penalties derived from center distance, aspect ratio, and the area of the minimum enclosing box. For instance, DIoU [12] explicitly penalizes the normalized distance between the centroids of the predicted and ground truth boxes to accelerate convergence. CIoU [12] expands upon this by incorporating a term for aspect ratio consistency. Subsequent variants, such as SIoU [13] and EIoU [14], introduce angle-aware components and reformulate aspect ratio penalties to enhance optimization stability. Concurrently, alternative research directions substitute overlap-based measures with metrics grounded in distance or distributional similarity, such as the Wasserstein distance [17,18]. These approaches are particularly advantageous as they preserve informative gradients even in non-overlapping scenarios.

However, a crucial limitation remains: most of these formulations continue to supervise the bounding box as a single, holistic entity. Specifically, they aggregate error signals based on global properties such as area, center, or aspect ratio. This structure inherently renders precise corner alignment an implicit learning target rather than an explicit optimization objective. Consequently, the regression head must infer the correct vertex positions indirectly from these holistic cues. This limitation is particularly evident in challenging detection scenarios, characterized by dense layouts, and pronounced scale variation. Datasets such as VisDrone2019 [19] exemplify these conditions. Under such circumstances, even a well-centered box with an appropriate aspect ratio can experience substantial IoU degradation due to misaligned corners. Therefore, corner misalignment emerges as a major source of localization error and a key factor contributing to IoU instability, especially for elongated or irregularly shaped objects.

To address this gap, we introduce ATDIoU, a vertex arctangent differential distance loss that explicitly supervises the four corresponding vertices of the predicted and ground truth boxes. Our core insight is that by directly targeting the primary source of localization error, the vertices, we can deliver a more focused and effective supervisory signal.

**Figure 1:**
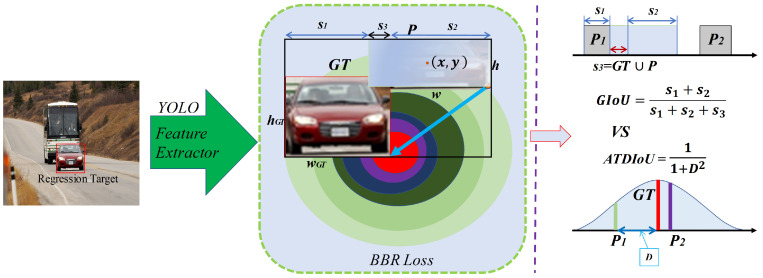
Illustration of the ATDIoU computation. $S_{1}$, $S_{2}$, and $S_{3}$ denote the regions indicated by the arrows; their areas are computed from the widths and heights of the corresponding bounding boxes. *D* denotes the distance between the corresponding corner points of the two bounding boxes.

The method maps each vertex offset to a two-dimensional arctangent differential (ATD) similarity, as shown in Figure 1. This mapping yields a response that is bounded, smooth, and monotonic. Furthermore, the response remains sensitive and approximately linear for small deviations, which is crucial for promoting precise corner alignment and achieving high IoU thresholds. Conversely, the function saturates gracefully for large deviations. This property prevents unbounded penalties and ensures stable, non-zero gradients even in non-overlapping cases, a characteristic that is particularly beneficial during the initial training stages. Finally, the four vertex similarities are aggregated to form a robust localization signal that suppresses corner drift, maintains informative gradients throughout training, and reduces sensitivity to noisy offsets.

We integrated the proposed ATDIoU into the YOLOv6 [2] framework and evaluated its performance on PASCAL VOC [20,21] and the challenging VisDrone2019 [19] datasets. The empirical results unmistakably demonstrate consistent and substantial improvements in mean average precision (mAP) over representative IoU-based losses, including the recent MPDIoU [15] and WIoU [16]. These findings indicate that explicit vertex supervision, when paired with a carefully designed arctangent differential mapping, provides an effective and complementary alternative to overlap-centric objectives, thereby offering a promising direction for enhancing BBR precision.

Our contributions are summarized as follows:

1. We introduce ATDIoU, an arctangent-differential loss for BBR that augments IoU with corner-wise distance supervision. Unlike overlap-only or center-distance objectives, ATDIoU explicitly constrains the box geometry by penalizing the offsets of the corresponding top-left and bottom-right corners through a bounded arctangent-differential mapping, improving localization stability.
2. We provide empirical analyses of gradient behavior and optimization dynamics, showing that the arctangent-differential mapping yields smoother, non-saturating gradients under localization errors, which helps reduce box drift during training.
3. Extensive experiments verify that integrating ATDIoU into a modern detector, such as YOLOv6, YOLOV10, achieves consistent mAP gains on both the PASCAL VOC and the VisDrone2019 datasets.

## 2. Related Work

### 2.1. IoU-Based BBR Losses

The evolution of BBR losses has been largely dominated by the IoU metric and its numerous extensions, owing to its intuitive geometric interpretation and direct alignment with evaluation metrics. The standard IoU loss, defined as $L_{IoU}=1-IoU$, suffers from vanishing gradients when two boxes do not overlap. To address this, GIoU [11] extends IoU by penalizing the area of the smallest enclosing box, thereby ensuring non-zero gradients when the boxes do not overlap. Nevertheless, the enclosing-box area is a global scalar that aggregates multiple geometric discrepancies into a single quantity. Consequently, different misalignment modes can yield similar enclosing areas, making the resulting gradients less informative for vertex-level correction, particularly when the dominant error is corner offset rather than pure center displacement. However, ATDIoU addresses this limitation by introducing vertex-direction supervision, which decomposes the mismatch into deviations of corresponding vertices. The proposed arctangent-differential mapping yields a bounded, continuous penalty, preventing unstable updates caused by extreme outliers while retaining a usable optimization signal in non-overlapping regimes. Importantly, because the penalty is defined at the vertex level, the gradients carry explicit directional information, encouraging each predicted corner to move toward its ground truth counterpart.

The subsequent development of DIoU [12] marked a significant step forward by incorporating the normalized central distance between the two boxes. This directly minimizes the distance between centers, leading to much faster convergence. CIoU [12] built upon DIoU by adding a consistency term for the aspect ratio, further improving the accuracy of the regressed boxes. Despite these improvements, both DIoU and CIoU can be influenced by the aspect ratio term in ways that do not always perfectly correlate with IoU improvement.

More recent variants have sought to incorporate additional geometric factors. SIoU [13] rethinks the cost function by introducing an angle cost that aims to minimize the angle between the central line and the x- or y-axis. This modification potentially leads to faster convergence and better final performance. EIoU [14] decomposes the loss into distance, overlap, and aspect ratio components, and reformulates the aspect ratio term to be more stable during training. WIoU [16] introduces a dynamic focusing mechanism that moderates the penalty on low-quality examples, thereby improving overall generalization. Another recent innovation, MPDIoU [15], leverages the minimum point distance to simplify the similarity calculation while maintaining effectiveness. These methods demonstrate the ongoing effort to refine the geometric supervision of BBR.

Nevertheless, IoU-family losses remain fundamentally overlap-centric. This design leads to two limitations. First, gradients may be brittle when boxes overlap only marginally or are disjoint, as the primary supervisory signal remains tied to the intersection area. Second, slight shifts near box boundaries can still induce unstable updates, because the IoU value is highly sensitive to edge contacts. These issues are especially pronounced for crowded scenes, where precise vertex placement is critical to disentangling adjacent objects.

### 2.2. Object Detection

Modern object detection frameworks are commonly categorized into three major families: two-stage detectors, one-stage detectors, and Transformer-based detectors. The evolution of these frameworks has correspondingly shaped the requirements and design of BBR losses.

Two-stage detectors, pioneered by the R-CNN series and subsequently refined into Faster R-CNN [5], first generate region proposals and then perform classification and bounding box refinement on those proposals. This paradigm typically achieves robust accuracy, benefitting from multi-scale feature representations, such as Feature Pyramid Networks (FPN) [22,23]. Region-of-interest (RoI) pooling or alignment operations facilitate refined feature extraction for each proposal, which underscores the importance of precise BBR within the second-stage regression module.

One-stage detectors streamline the pipeline by directly regressing bounding boxes from dense sampling locations across the image, thereby prioritizing computational speed and architectural simplicity. Anchor-based methods (e.g., SSD [24], RetinaNet [25], and the YOLO family [2,6,26]) rely on predefined anchor boxes. Techniques such as focal loss [25] were introduced to alleviate the extreme foreground–background class imbalance inherent in dense prediction. In parallel, advances in feature pyramid fusion modules, including PANet [27] and BiFPN [28], have substantially enhanced object detection by improving the bidirectional flow of spatial and semantic information. More recent anchor-free detectors (e.g., CornerNet [29], FCOS [30]) obviate the need for handcrafted anchor designs by predicting keypoints (e.g., corners) or center–size pairs. Further progress has been driven by dynamic label assignment strategies and quality-aware prediction heads, which improve both training stability and detection accuracy. Given that these models rely on direct regression without a secondary refinement stage, the choice of BBR loss is particularly paramount.

Transformer-based detectors such as DETR [31] have recently reconceptualized object detection as a set prediction task, thus eliminating many manual components, including anchors and non-maximum suppression. Although promising, the original DETR exhibits slow convergence. Subsequent variants like Deformable-DETR [32] and DINO [33] ameliorate this deficiency by introducing deformable attention mechanisms and enhanced denoising training, which enables faster convergence and stronger multi-scale reasoning. Real-time extensions such as RT-DETR [31] further demonstrate ongoing efforts to render Transformer-based detection viable for industrial deployment. The end-to-end nature of these models also imposes specific requirements on the regression loss, which must maintain stability and effectiveness within a complex, set-based optimization landscape.

### 2.3. Vertex-Aware Localization

While the corners of a bounding box represent its most definitive geometric features, their explicit and direct supervision within loss functions remains relatively underexplored, particularly in mainstream detection frameworks. Corner-based supervision is a recognized concept in keypoint-style detectors such as CornerNet [29], which directly predicts heatmaps for the top-left and bottom-right corners. However, this approach pertains to a specific detection paradigm and does not readily translate into a general BBR loss suitable for coordinate-based regression.

In standard regression heads, vertex alignment is typically an implicit target. Losses such as Smooth L1, when applied to box parameters, influence corner placement only indirectly. Although some methods encourage edge alignment through auxiliary constraints, most BBR objectives still aggregate localization errors at the holistic box level, relying on metrics such as overlap, center distance, or aspect ratio. Consequently, explicit modeling of vertex-to-vertex relationships and direct optimization of corner distances have remained relatively niche.

A parallel line of research has explored alternatives to IoU by shifting towards distance-based or distribution-based metrics. For instance, the Normalized Wasserstein Distance (NWD) [17] has been proposed for tiny object detection because it is less sensitive to minor spatial shifts than IoU. Similarly, the Gaussian Wasserstein Distance [18] and the Kullback–Leibler Divergence have been investigated for rotated object detection, where IoU computation becomes more complex and unstable. These works underscore the potential of direct distance measures in addressing the shortcomings of overlap-centric approaches.

Our proposed ATDIoU lies at the intersection of these ideas. It draws inspiration from the benefits of direct geometric supervision seen in keypoint-based methods and from the robustness of distance-based losses. Crucially, it implements these principles in a way that is compatible with mainstream coordinate-based detectors. ATDIoU computes a bounded similarity between corresponding vertices using an arctangent differential mapping. By supervising the specific geometric elements, the corners, that most directly contribute to edge and corner drift, ATDIoU provides a more targeted and effective localization signal that effectively complements existing holistic losses.

## 3. Methods

In this section, we detail the design philosophy and mathematical formulation of ATDIoU. First, to validate the effectiveness of the proposed regression behavior, we construct simulated experiments that examine scale, and aspect ratio relationships. We then introduce the arctangent-differential function (ATD), which serves as the core mathematical component of our approach. Finally, we formally define the ATDIoU loss, which computes distance similarity between predicted and ground truth boxes through vertex-based arctangent differential mappings, thereby enabling more precise and reliable localization supervision.

### 3.1. Simulation Experiment

**Figure 2:**
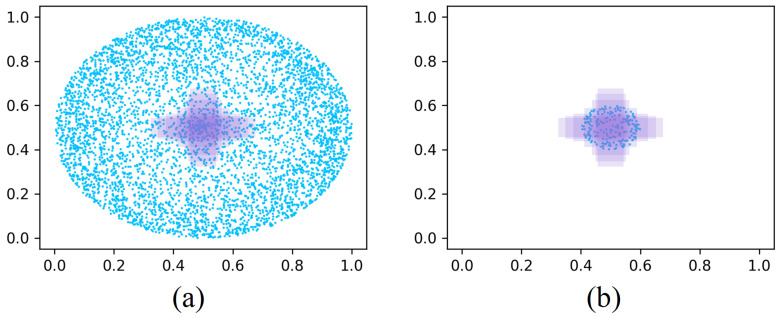
Anchor locations and target boxes in the simulation. (**a**) all cases. (**b**) main case.

We further evaluate the regression behavior of ATDIoU using simulation experiments [12]. The setup spans the principal relationships between bounding boxes distance, scale, and aspect ratio. Specifically, seven unit-area target boxes (areas fixed at 1) with aspect ratios of 1:4, 1:3, 1:2, 1:1, 2:1, 3:1, and 4:1 are defined. To simplify the setup, the centers of the target boxes are fixed and evenly distributed across 6000 locations, as depicted in Figure 2. (I) Distance: For each location, we sample points uniformly within a circle of radius 0.3 centered at (0.5,0.5); the seven anchor scales and seven aspect ratios are uniformly instantiated within this region, yielding both overlapping and non-overlapping cases. (II) Scale: For each location, the anchor-box area is set to one of 0.50, 0.67, 0.75, 1.00, 1.33, 1.50, 2.00. (III) Aspect ratio: For each location and scale, the seven aspect ratios listed above are used, matching the target box settings. In total, we instantiate 6000×7×7×7=2,058,000 regression cases. The total error metric (*E*) used for evaluation is defined as follows:

(1) $$E^{i}=\sum_{n=1}^{6000}\sum_{t\in x,y,w,h}\left|B_{t,n}^{\left(i\right)}-B_{t,n}^{gt}\right|,$$

where $B_{t,n}^{i}$ denotes the *t*-th parameter of the current box for sample *n* at iteration *i*, and $B_{t,n}^{gt}$ is the corresponding ground truth. Training uses stochastic gradient descent (SGD) with a step learning rate scheduler (initial learning rate 0.1; step size 80) for 150 epochs.

### 3.2. Arctangent Differential Function

**Figure 3:**
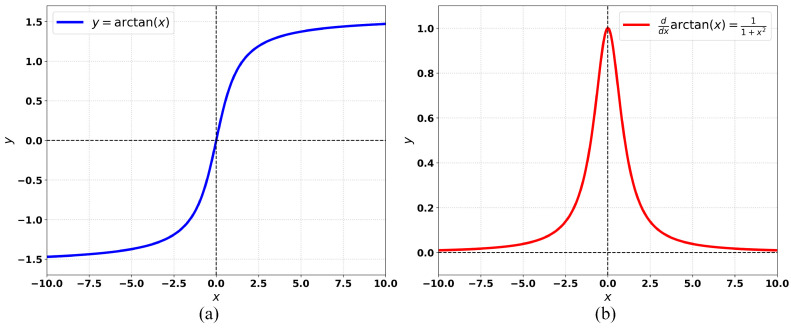
Cartesian coordinate systems for the arctangent and arctangent-differential functions. (**a**) arctangent function. (**b**) arctangent-differential function.

The arctangent-differential function has diverse applications in mathematics and engineering [34], as illustrated in Figure 3a. In deep learning, it can serve as a loss component to improve gradient flow and adapt to data distributions, thereby accelerating convergence. This formulation helps models capture complex patterns and enhances generalization. For object detection, using the arctangent-differential function as a BBR loss enables more accurate localization of bounding boxes, improving both accuracy and robustness of the detector.

Inspired by the properties of the arctangent, shown in Figure 3, we propose a BBR loss based on its derivative (the arctangent-differential function). The arctangent is defined as

(2) f(x)=arctan(x),

with derivative

(3) $$f^{\prime }\left(x\right)=\frac{1}{1+x^{2}},$$

where x∈R is the input, f(x) denotes the arctangent, and $f^{\prime }\left(x\right)$ its derivative. The graphs of f(x) and $f^{\prime }\left(x\right)$ are shown in Figure 3a and Figure 3b, respectively.

### 3.3. ATDIoU

**Figure 4:**
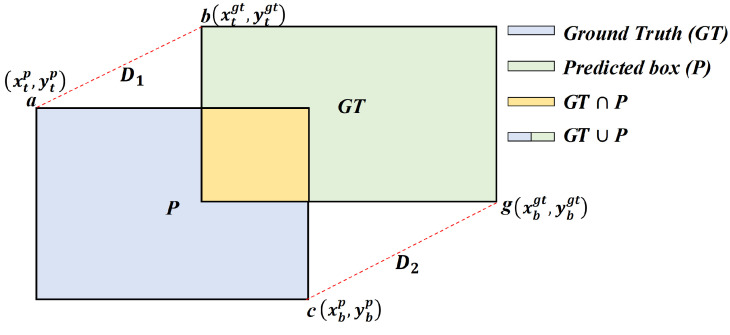
IoU and arctangent differential metrics. IoU between ground truth (GT) and predicted box (*P*). Arctangent differential distance-similarity computed for point pairs (a,b) and (c,g).

ATDIoU measures the similarity between a predicted box and its ground truth by mapping vertex-wise Euclidean offsets to a bounded 2D arctangent-differential score, as shown in Figure 4. After feature extraction, the detector outputs multiple predicted boxes. These boxes are compared with the ground truth using a BBR loss. ATDIoU emphasizes near ground truth predictions by assigning greater optimization weight to boxes that are already close to the target, thereby accelerating the refinement of high-quality predictions and speeding up bounding box convergence. Specifically, after feature extraction by the neural network, a 4-D output (x,y,w,h) is produced. As shown in Figure 1, (x,y) denotes the center of the predicted box (*P*), and (w,h) denotes its width and height. The top-left corner *a* and bottom-right corner *c* of *P* are

(4) $$a=\left(x-\frac{1}{2}w;y-\frac{1}{2}h\right),\;c=\left(x+\frac{1}{2}w;y+\frac{1}{2}h\right).$$

We denote their coordinates as $\left(x_{t}^{p},y_{t}^{p}\right)$ and $\left(x_{b}^{p},y_{b}^{p}\right)$, respectively. Let the corresponding ground truth corners be $b=(x_{t}^{gt},y_{t}^{gt})$ for the top-left and $g=(x_{b}^{gt},y_{b}^{gt})$ for the bottom-right.

The IoU between a ground truth (GT) and a predicted box (*P*) (Algorithm 1) is defined as

(5) $$IoU=\frac{\left|GT\cap P\right|}{\left|GT\cup P\right|}.$$

Let *a* and *c* denote the top-left and bottom-right corners of the ground truth, and *b* and *g* the corresponding corners of the predicted box. Define the squared Euclidean distances

(6) $$w_{t}^{2}=∥x_{t}^{gt}-x_{t}^{p}{∥}_{2}^{2}+{∥y_{t}^{gt}-y_{t}^{p}∥}_{2}^{2},$$

(7) $$w_{b}^{2}=∥x_{b}^{gt}-x_{b}^{p}{∥}_{2}^{2}+{∥y_{b}^{gt}-y_{b}^{p}∥}_{2}^{2},$$

and the arctangent differential weights

(8) $$D_{t}=\frac{\partial }{\partial x,y}\arctan\left(w_{t}^{2}\right)=\frac{1}{1+w_{t}^{2}},$$

(9) $$D_{b}=\frac{\partial }{\partial x,y}\arctan\left(w_{b}^{2}\right)=\frac{1}{1+w_{b}^{2}}.$$

ATDIoU is then defined as

(10) $$ATDIoU=IoU-\alpha D_{t}-\beta D_{b},$$

where α and β are tuning parameters (both set to 0.03 in our experiments). Consequently, the ATDIoU loss is

(11) $$L_{ATDIoU}=1-ATDIoU.$$

**Algorithm 1** ATDIoU as BBR loss  1:**input:**   Predicted box *P* and ground truth GT coordinates. $P=(x_{1}^{p},y_{1}^{p},x_{2}^{p},y_{2}^{p})$, $GT=\left(x_{1}^{g},y_{1}^{g},x_{2}^{g},y_{2}^{g}\right)$:  2:**output:** $L_{ATDIoU}$.  3:For the predicted box B, ensuring $x_{2}^{p}>x_{1}^{p}$ and $y_{2}^{p}>y_{1}^{p}.$  4:$w_{1}^{2}={\left(x_{1}^{p}-x_{1}^{g}\right)}^{2}+{\left(y_{1}^{p}-y_{1}^{g}\right)}^{2}$  5:$w_{2}^{2}={\left(x_{2}^{p}-x_{2}^{g}\right)}^{2}+{\left(y_{2}^{p}-y_{2}^{g}\right)}^{2}$  6:Calculating area of $S^{g}$: $S^{g}=\left(x_{2}^{g}-x_{1}^{g}\right)\left(y_{2}^{g}-y_{1}^{g}\right)$  7:Calculating area of $S^{p}$: $S^{p}=\left(x_{2}^{p}-x_{1}^{p}\right)\left(y_{2}^{p}-y_{1}^{p}\right)$  8:Calculating intersection *i* between $S^{g}$ and $S^{p}$: $\begin{array}{c} x_{1}^{i}=max\left(x_{1}^{p},x_{1}^{g}\right),x_{2}^{i}=min\left(x_{2}^{p},x_{2}^{g}\right)\;y_{1}^{i}=max\left(y_{1}^{p},y_{1}^{g}\right),y_{2}^{i}=min\left(y_{2}^{p},y_{2}^{g}\right)\;\;i=\left\{\begin{array}{cc} \left(x_{2}^{i}-x_{1}^{i}\right)\left(y_{2}^{i}-y_{1}^{i}\right), & if\;x_{2}^{i}>x_{1}^{i},y_{2}^{i}>y_{1}^{i}\\ 0, & otherwise. \end{array}\right. \end{array}$  9:$IoU=\frac{i}{U},\;\mathrm{where}\;U=S^{g}+S^{p}-i$10:$ATDIoU=IoU-\frac{\alpha }{1+w_{1}^{2}}-\frac{\beta }{1+w_{2}^{2}}$11:$L_{ATDIoU}=1-ATDIoU$

## 4. Experiments

To thoroughly evaluate the performance of ATDIoU in object detection scenarios, this section presents extensive comparative experiments. We integrated the proposed loss function into the YOLOv6L framework and conducted evaluations on two datasets: PASCAL VOC and VisDrone2019. Through detailed descriptions of the experimental setup and comprehensive quantitative analyses, we perform a side-by-side comparison of ATDIoU with several mainstream BBR loss functions. In addition, we further examine the robustness and efficacy of our method.

### 4.1. Datasets

The PASCAL Visual Object Classes (PASCAL VOC) [20,21] benchmark dataset holds considerable significance in the field of computer vision. In this work, we utilize a combination of the VOC 2007 and VOC 2012 versions. The dataset encompasses 20 common object categories, which can be broadly grouped into four classes: humans, animals (e.g., birds, cats, dogs), vehicles (e.g., aeroplanes, bicycles, cars), and indoor objects (e.g., bottles, chairs, sofas). It provides rich annotation types, including image-level classification labels, bounding boxes for object detection, and semantic segmentation masks. Owing to its high annotation quality, well-characterized scenes, and relatively balanced category distribution, PASCAL VOC has become a foundational benchmark for assessing the core performance of object detection algorithms. It is widely used to evaluate a model’s feature extraction capability and its generalization ability under typical ground-level visual conditions.

The VisDrone2019 [19] dataset is a large-scale benchmark designed for unmanned aerial vehicle (UAV) visual analysis, targeting the unique challenges of object detection and tracking from aerial viewpoints. It comprises tens of thousands of static images and video sequences captured by multiple UAV platforms across diverse urban environments, weather conditions (including clear, overcast, and nighttime scenes), and illumination settings, with more than 2.6 million manually annotated bounding boxes. The dataset includes ten common categories of ground and traffic targets, such as pedestrians, vehicles, lorries, and buses. Unlike ground-level datasets such as PASCAL VOC, VisDrone2019 presents a distinct bird’s-eye perspective and is characterized by extremely small object scales, high object density, complex and dynamic backgrounds, and severe occlusion. As a result, VisDrone2019 has become an essential benchmark for evaluating the performance of deep learning models in small object detection, dense scene understanding, and robustness to complex aerial environments.

### 4.2. Experimental Setup

YOLOv6 [2] is a single-stage object detection framework developed by Meituan’s Visual Intelligence Department. Unlike traditional YOLO variants, it abandons the conventional anchor-based paradigm in favor of an anchor-free design, thereby eliminating the need for extensive anchor-box hyperparameter tuning and reducing computational overhead. At the architectural level, YOLOv6 introduces the EfficientRep backbone, which leverages reparameterization techniques. During training, this backbone adopts a multi-branch structure to enhance feature extraction and improve gradient backpropagation efficiency. At inference time, the branches are merged into a mathematically equivalent single-path structure, which increases hardware computational density and improves memory access efficiency. In addition, YOLOv6 employs an efficient decoupled head that separates classification and regression tasks. Together with advanced label-assignment strategies, quantization-aware training, and knowledge distillation, these design choices further maximize the model’s performance potential.

All experiments were conducted on two NVIDIA RTX 3090 (NVIDIA, Santa Clara, CA, USA) servers running PyTorch 1.13 with CUDA 11.6 on Ubuntu 20.04. We fine-tuned the YOLOv6L pretrained model using a batch size of 48 for 150 epochs. The optimizer used was stochastic gradient descent (SGD) with a learning rate of 0.0032, momentum of 0.843, and weight decay of 0.00036. Data augmentation techniques included rotation, translation, flipping, image decay, and Mosaic augmentation. All experiments were conducted using identical parameter settings.

### 4.3. Metrics

To comprehensively evaluate the performance of the object detection models, we adopt the standard MS COCO evaluation protocol, which uses mean average precision (mAP) as its primary metric across multiple IoU thresholds and object scales. Specifically, mAP50 and mAP75 denote detection accuracy at IoU thresholds of 0.50 and 0.75, reflecting both lenient and strict localization requirements. The overall mAP95 (i.e., mAP@[0.50:0.95]) is computed by averaging the results over the IoU threshold set T=0.50,0.55,…,0.95 with an interval of 0.05:

(12) $$mAP95=mAP@\left[0.50:0.95\right]=\frac{1}{\left|T\right|}\sum_{t\in T}\left(\frac{1}{C}\sum_{i=1}^{C}AP_{i,t}\right),\;\left|T\right|=10.$$

To analyze performance at different object scales, we report the standard COCO metrics: $mAP_{s}$ for small objects (area $<32^{2}$ pixels), $mAP_{m}$ for medium objects ($32^{2}\leq \mathrm{area}<96^{2}$ pixels), and $mAP_{l}$ for large objects (area $\geq 96^{2}$ pixels).

All metrics are derived from Precision (*P*) and Recall (*R*), defined as:

(13) $$P=\frac{TP}{TP+FP},\;R=\frac{TP}{TP+FN}.$$

For each category, the Average Precision (AP) is computed as the area under its precision–recall (*P*–*R*) curve:

(14) $$AP=\int_{0}^{1}P\left(R\right),dR.$$

The overall category-level mAP is then obtained by averaging AP across all *C* categories:

(15) $$mAP=\frac{1}{C}\sum_{i=1}^{C}AP_{i}.$$

### 4.4. Experimental Analysis

To comprehensively validate the effectiveness of ATDIoU, we conduct an in-depth evaluation combining quantitative metrics with qualitative visualization results. The experimental findings demonstrate that ATDIoU not only performs strongly on general detection tasks but also exhibits notable advantages in small object detection, dense scenes, and high-precision localization scenarios.

Firstly, the quantitative results on PASCAL VOC dataset (Table 1) highlight ATDIoU’s superior localization accuracy. ATDIoU achieves an overall mAP of 0.701, outperforming the second-best DIoU by 1.1 percentage points. Notably, on the mAP75 metric, which reflects a model’s capability under stringent localization requirements, ATDIoU reaches 0.767, substantially surpassing mainstream loss functions such as GIoU and DIoU.

This improvement stems from ATDIoU’s design philosophy: by explicitly supervising the offsets of all four bounding box vertices rather than relying solely on holistic overlap, it more effectively suppresses corner drift. Consequently, ATDIoU performs consistently better at higher IoU thresholds. Moreover, ATDIoU achieves the best performance across all scale metrics, $mAP_{s}$, $mAP_{m}$, and $mAP_{l}$. These gains indicate that the smooth and bounded nature of the arctangent-differential function enables stable gradient flow and robust regression behavior across objects of varying scales.

Secondly, the experimental results on the VisDrone2019 dataset (Table 2) further demonstrate ATDIoU’s robustness in dense small object scenarios. On the highly challenging $mAP_{s}$ metric, ATDIoU obtains a score of 0.135, outperforming both EIoU and SIoU. Since small objects are extremely sensitive to pixel-level positional deviations, traditional IoU-based losses often exhibit unstable gradients. ATDIoU, however, leverages the high sensitivity of the arctangent function near zero to perform fine-grained calibration for small deviations, substantially enhancing recall for tiny objects. Additionally, ATDIoU achieves the best performance on the $mAP_{m}$ metric with a score of 0.377, confirming its ability to provide more discriminative and reliable supervision for small-to-medium objects in complex aerial scenes.

To further validate the generalization capability of the ATDIoU loss function across diverse detection frameworks, we integrated it into the YOLOv10m model and conducted comparative experiments on the VisDrone2019 dataset. As detailed in Table 3, ATDIoU demonstrated superior performance across all four key metrics, precision (0.439), recall (0.353), *mAP*@50 (0.343), and *mAP*@95 (0.185), surpassing mainstream loss functions such as EIoU and GIoU. These findings align with the model’s excellent performance within the YOLOv6 framework, collectively confirming the effectiveness and robustness of the proposed method.

**Figure 5:**
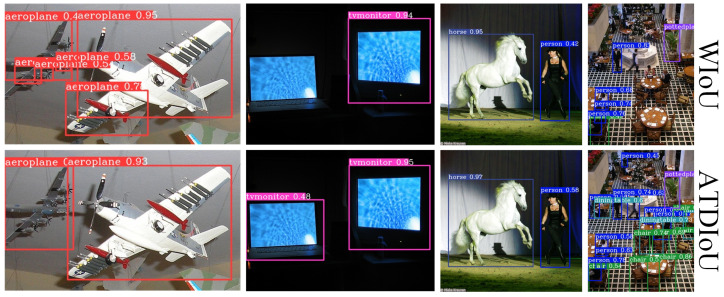
Inference performance across BBR losses on small and low-contrast targets.

**Figure 6:**
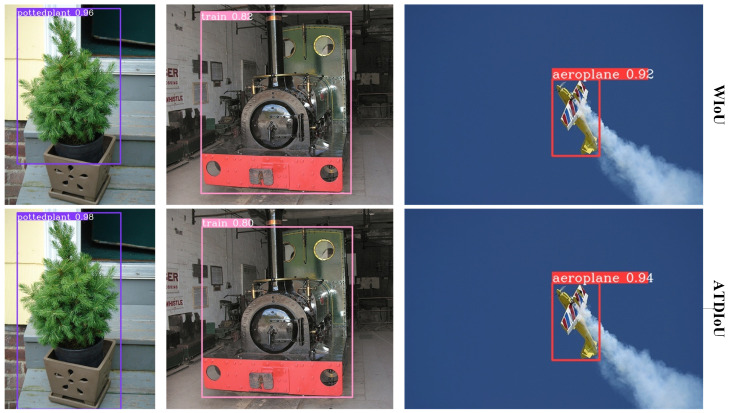
Inference performance on PASCAL VOC across BBR losses. ATDIoU achieves more accurate localization and higher detection confidence than alternative losses.

**Figure 7:**
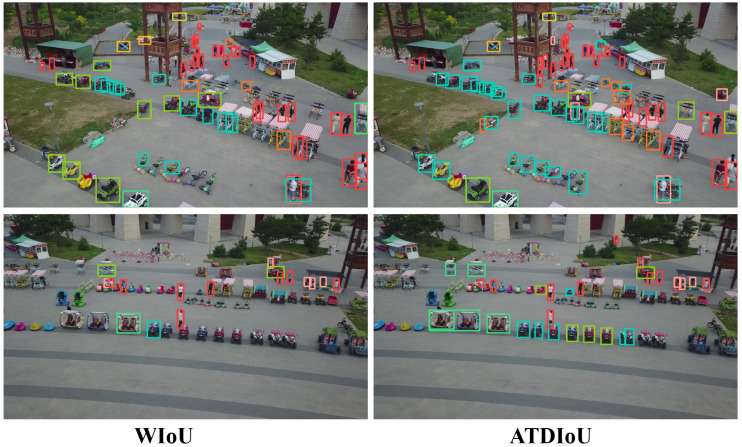
Inference performance across BBR losses on the VisDrone2019.

Finally, the qualitative inference results (Figure 5, Figure 6 and Figure 7) provide intuitive confirmation of the quantitative improvements. As shown in Figure 6, in general scenes, ATDIoU not only produces bounding boxes that align more accurately with object boundaries but also increases detection confidence (e.g., aircraft confidence rising from 0.92 under WIoU to 0.94). This suggests that precise localization feedback assists the classification branch in learning more discriminative features.

In the low-contrast and occluded scenarios illustrated in Figure 5, ATDIoU effectively mitigates missed detections because the arctangent differential distribution provides stable gradient propagation even in cases of blurred boundaries or non-overlapping boxes. Furthermore, in dense scenarios such as those shown in Figure 7, ATDIoU exhibits strong robustness against mutual interference, successfully separating closely adjacent objects and avoiding the optimization ambiguities commonly encountered with overlap-centric loss functions.

**Figure 8:**
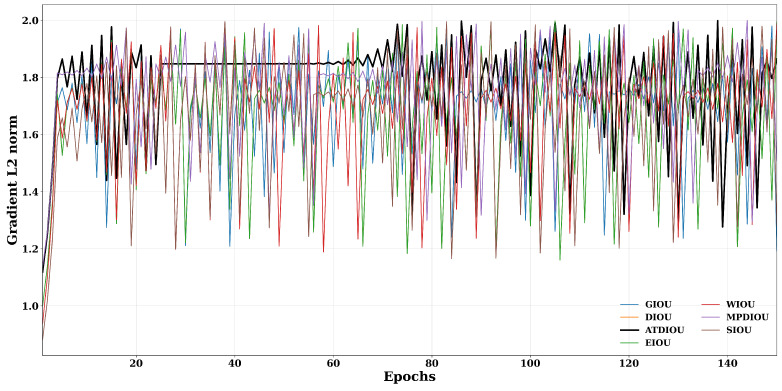
Gradient update performance of BBR losses.

As shown in the gradient norm curves in Figure 8 and the quantitative statistics in Table 4, different BBR losses exhibit distinct optimization behaviors. Overall, all losses maintain relatively large gradient $L_{2}$ norms (primarily in the range of 1.6–1.9), indicating that none of them suffers from severe gradient vanishing. However, both the gradient magnitude and its stability vary significantly across methods. ATDIoU consistently stays near the upper envelope of all curves, achieving the highest average gradient norm (mean=1.7746) and the smallest standard deviation and coefficient of variation (std=0.1853, CV=0.1044). This superior performance leads to the best composite score (1.6069). These findings imply that ATDIoU provides not only richer gradient information but also a more stable optimization signal throughout training. MPDIoU ranks second, with a slightly lower mean gradient and higher CV, suggesting good but somewhat more oscillatory gradients compared with ATDIoU. In contrast, conventional IoU-based losses, such as GIoU, WIoU, DIoU/EIoU, and SIoU, present lower gradient means (ranging from 1.67–1.70) and noticeably larger CV values (up to 0.1431 for SIoU). This corresponds to more frequent and sharper fluctuations visible in the curves. Collectively, these results demonstrate that ATDIoU achieves a superior balance between gradient magnitude and stability, thereby offering a stronger and more reliable optimization driving force for BBR on VisDrone2019.

In summary, by introducing vertex-level differential supervision, ATDIoU effectively overcomes the limitations of traditional IoU-based losses—namely, their insufficient sensitivity to positional bias and their unstable gradient behavior at extreme scales.

## 5. Discussion

Although ATDIoU consistently improves localization accuracy, two practical considerations should be noted when deploying it as a training objective. First, ATDIoU adds a small number of elementwise operations (arctangent and division) on top of IoU computation, which slightly increases training-time cost but does not introduce extra parameters or change the detector’s inference-time computation. Second, ATDIoU involves two weights (α and β). In our experiments, we fixed α = β = 0.03 across both PASCAL VOC and VisDrone2019, but transferring to other detectors or domains may benefit from modest tuning; a practical starting point is to keep α and β equal and adjust them based on the localization–recall trade-off.

Future work will focus on (i) reducing training-time overhead via efficient approximations of the arctangent-differential term, (ii) designing adaptive weighting to eliminate manual selection of α and β when transferring across datasets, and (iii) extending ATDIoU to broader localization settings (e.g., dense detection regimes and other box parameterizations) and combining it with complementary objectives for further gains.

## 6. Conclusions

The ATDIoU loss exhibits notable advantages in object detection. Compared with MPDIoU, it increases AP50 by 1.4% on the PASCAL VOC and by 0.7% on the VisDrone dataset. The ATDIoU can be attributed to the adopted arctangent differential formulation, which helps mitigate bounding box drift and reduce sensitivity to localization errors. Consequently, ATDIoU encourages the model to learn object locations more effectively, thereby improving overall detection performance. Nonetheless, ATDIoU may face practical challenges, including increased computational overhead and sensitivity to hyperparameters. Future work could focus on algorithmic optimizations to reduce compute cost, systematic studies of applicability across diverse vision tasks, and combinations with complementary loss functions to address remaining limitations.

## Figures and Tables

**Table 1:** Performance of BBR losses on PASCAL VOC, including the mean, variance, and 95% confidence interval of ATDIoU.

	${\mathrm{mAP}}_{s}$	${\mathrm{mAP}}_{m}$	${\mathrm{mAP}}_{l}$	mAP95	mAP50	mAP75
EIoU	0.274±0.002	0.572±0.005	0.788±0.002	0.689±0.001	0.873±0.003	0.747±0.003
GIoU	0.267±0.003	0.570±0.003	0.790±0.001	0.693±0.002	0.873±0.004	0.755±0.004
SIoU	0.268±0.003	0.559±0.002	0.786±0.003	0.685±0.003	0.872±0.002	0.750±0.000
MPDIoU	0.265±0.002	0.563±0.002	0.787±0.001	0.684±0.003	0.871±0.002	0.749±0.001
DIoU	0.266±0.001	0.566±0.002	0.787±0.002	0.690±0.000	0.878±0.001	0.746±0.003
WIoU	0.263±0.001	0.562±0.003	0.787±0.003	0.692±0.004	0.878±0.004	0.755±0.002
ATDIoU	0.283±0.002	0.57±0.003	0.798±0.001	0.70±0.000	0.886±0.003	0.767±0.000
95%CI	0.283±0.005	0.57±0.007	0.798±0.002	0.70±0.000	0.886±0.007	0.767±0.000

**Table 2:** Performance of BBR losses on the VisDrone2019, including the mean, variance, and 95% confidence interval of ATDIoU.

	${\mathrm{mAP}}_{s}$	${\mathrm{mAP}}_{m}$	${\mathrm{mAP}}_{l}$	mAP95	mAP50	mAP75
EIoU	0.130 ± 0.0009	0.374 ± 0.0041	0.614 ± 0.0030	0.238 ± 0.0011	0.396 ± 0.0031	0.238 ± 0.0028
GIoU	0.126 ± 0.0035	0.373 ± 0.0051	0.631 ± 0.0009	0.239 ± 0.0024	0.396 ± 0.0035	0.246 ± 0.0037
SIoU	0.134 ± 0.0031	0.364 ± 0.0033	0.621 ± 0.0035	0.236 ± 0.0034	0.397 ± 0.0019	0.240 ± 0.0009
MPDIoU	0.129 ± 0.0017	0.367 ± 0.0029	0.621 ± 0.0010	0.234 ± 0.0031	0.395 ± 0.0020	0.239 ± 0.0012
DIoU	0.130 ± 0.0028	0.372 ± 0.0029	0.611 ± 0.0020	0.236 ± 0.0012	0.397 ± 0.0011	0.236 ± 0.0036
WIoU	0.131 ± 0.0023	0.373 ± 0.0030	0.621 ± 0.0026	0.244 ± 0.0035	0.403 ± 0.0040	0.245 ± 0.0019
ATDIoU	0.135 ± 0.0018	0.377 ± 0.0006	0.626 ± 0.0027	0.242 ± 0.0005	0.404 ± 0.0026	0.247 ± 0.0002
95%CI	0.135 ± 0.0045	0.377 ± 0.0015	0.626 ± 0.0068	0.242 ± 0.0012	0.404 ± 0.0065	0.247 ± 0.0005

**Table 3:** Performance of YOLOv10m integrated with BBR losses on the VisDrone2019.

	Precision	Recall	mAP50	mAP95
NWD	0.425	0.341	0.335	0.175
GWD	0.431	0.346	0.337	0.179
EIoU	0.430	0.348	0.339	0.182
GIoU	0.429	0.349	0.338	0.180
SIoU	0.426	0.343	0.333	0.178
DIoU	0.433	0.348	0.339	0.181
ATDIoU	0.439	0.353	0.343	0.185

**Table 4:** Statistical comparison of BBR losses: mean, standard deviation (Std), coefficient of variation (CV), and comprehensive score.

	Mean ↑	Std ↓	CV ↓	Score ↑
MPDIOU	1.7476	0.1890	0.1081	1.5771
GIoU	1.7016	0.1952	0.1147	1.5265
WIoU	1.6862	0.2172	0.1288	1.4938
DIoU	1.6757	0.2282	0.1362	1.4748
EIoU	1.6757	0.2282	0.1362	1.4748
SIoU	1.6678	0.2386	0.1431	1.4590
ATDIoU	1.7746	0.1853	0.1044	1.6069

## Data Availability

All datasets used in this study are publicly available and ethically compliant. There are no competing interests associated with the data. The code used and analyzed during the current study is available from the corresponding or first author upon reasonable request.
